# One-Pot Tandem Synthesis of 2-Arylquinazolines by a Multicomponent Cyclization Reaction

**DOI:** 10.3390/molecules181113860

**Published:** 2013-11-08

**Authors:** Leping Ye, Lin Yu, Lijun Zhu, Xiaodong Xia

**Affiliations:** The Second Affiliated Hospital and Yuying Children’s Hospital of Wenzhou Medical University, Wenzhou 325000, Zhejiang, China

**Keywords:** quinazolines, multicomponent, cyclization reaction, one-pot tandem synthesis

## Abstract

A series of 2-arylquinazolines have been synthesized in moderate to excellent yields by one-pot tandem reaction of (2-aminophenyl)methanols, aldehydes and ceric ammonium nitrate (CAN). The utility of this transformation was demonstrated by its compatibility with a wide range of functional groups. Thus, the method represents a simple and practical procedure to access 2-arylquinazolines.

## 1. Introduction

**Scheme 1:**
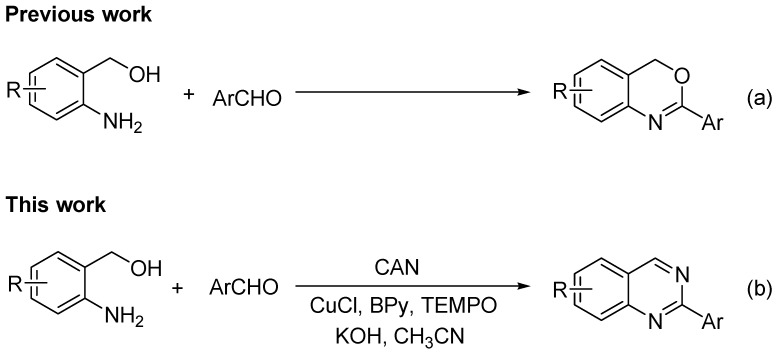
Reaction of (2-aminophenyl)methanols with aldehydes under different conditions.

Quinazolines and derivatives thereof are ubiquitous structural motifs that frequently occur in a wide variety of bioactive natural products such as prazosin [1], lapatinib [2], icotinib [3], *etc.* They possess a wide range of biological and pharmacological activities, including antibacterial [4], anti-inflammatory [5], antiplasmodial [6], antitumor [7], antimicrobial and antioxidant properties [8]. In addition, they have also been used as photochemotherapeutic agents [9], DNA-gyrase, JAK2, PDE5, and EGFR tyrosine kinase inhibitors [10] as well as CB2 receptor agonists [11]. Recently, Yang reported that substituted quinazolines possess novel potent and selective FLT3 inhibitory and anti-acute myeloid leukemia activities [12]. Consequently, in the past few years a number of synthetic methods to prepare these compounds have been described. Fu and co-workers reported that copper-catalyzed cascade reactions provide attractive and valuable routes for the construction of quinazoline derivatives via sequential Ullmann-type coupling and intramolecular cyclization [13]. Walton reported a microwave-promoted syntheses of quinazolines by the reaction of 2-(aminoaryl)alkanone *O*-phenyl oximes with aldehydes [14]. Tandem reaction of 2-aminobenzophenones with benzylic amines followed by C–H functionalization has also been reported [15,16,17,18]. Zhang reported a three-component synthesis of quinazoline derivatives using a low melting sugar–urea–salt mixture as a solvent [19]. In 2012, Beifuss and co-workers developed a copper-catalyzed coupling reaction of *o*-bromobenzylbromides and benzamidines for the synthesis of quinazolines in an aqueous medium [20]. The condensation reactions of 2-aminobenzylamines with aldehydes followed by subsequent oxidation with strong oxidants (e.g., 2,3-dichloro-5,6-dicyano-l,4-benzoquinone (DDQ) [21], MnO_2_ [22], and NaClO [23]) provide a conventional but simple method to synthesize quinazolines (Scheme 1(a)). However, these methods are hampered by the need to use a stoichiometric amount of a nonrenewable oxidant and the yields have not always been satisfactory. Recently, three improved methods have been reported for constructing quinazolines: (i) copper-catalyzed aerobic oxidative cyclization reaction of 2-(aminomethyl)benzenamines with aldehydes [24]; (ii) aerobic oxidative cyclization reaction of 2-(aminomethyl)benzenamines with aldehydes by a cooperative catalytic system of platinum/iridium alloyed nanoclusters and a dimeric catechol derivative [25]; (iii) iridium-catalyzed hydrogen transfer reaction of 2-(aminomethyl)benzenamines with aldehydes using styrene as a hydrogen acceptor [26].

Herein, we wish to report a new method for the synthesis of quinazolines by using a one-pot tandem reaction of (2-aminophenyl)methanols, aldehydes and ceric ammonium nitrate (CAN) (Scheme 1(b)).

## 2. Results and Discussion

We began our study by examining the reaction between (2-aminophenyl)methanol (**1a**) and benzaldehyde (**2a**) to obtain 2-phenyl-4*H*-benzo[*d*][1,3]oxazine (**4a**) and screen the optimal reaction conditions. Surprisingly, a trace amount of unexpected 2-phenylquinazoline (**3a**) was detected by GC/MS (EI) analysis during the process of performing the above condensation reaction in the presence of ceric ammonium nitrate (CAN). To the best of our knowledge, the synthesis of quinazolines using a one-pot tandem reaction of (2-aminophenyl)methanols, aldehydes and ceric ammonium nitrate has never been investigated before. In the present study, this unexpected reaction was thoroughly investigated. To realize the selective generation of **3a**, we initially ran a series of trial experiments in the presence of palladium catalysts by adjusting the reaction parameters. However, little-to-no product **3a** was detected. To our delight, after the catalyst was switched to a copper catalyst such as CuOTf, the desired product **3a** was isolated in 19% yield when the combination of KF and CH_3_CN was employed (Table 1, entry 1). Encouraged by this promising result, we further adjusted reaction parameters including copper catalysts, bases, and solvents.

**Table 1:** Optimization of the reaction conditions ^a^. 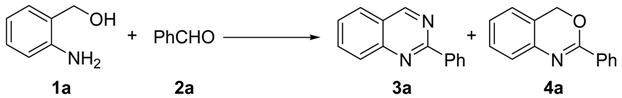

Entry	Cu sources	Base	Solvent	Yield (%) ^b^
1	CuOTf	KF	CH_3_CN	19
2	CuOTf	Li_2_CO_3_	CH_3_CN	32
3	CuOTf	CsOAc	CH_3_CN	17
4	CuOTf	K_2_CO_3_	CH_3_CN	45
5	CuOTf	K_3_PO_4_	CH_3_CN	14
6	CuOTf	*t*-BuOK	CH_3_CN	33
7	CuOTf	LiOH	CH_3_CN	42
8	CuOTf	NaOH	CH_3_CN	48
9	CuOTf	KOH	CH_3_CN	64
10	CuOTf	CsOH	CH_3_CN	68
11	CuBr	CsOH	CH_3_CN	82
12	CuCl	CsOH	CH_3_CN	91
13	Cu(OTf)_2_	CsOH	CH_3_CN	67
14	Cu(OAc)_2_	CsOH	CH_3_CN	56
15	CuBr_2_	CsOH	CH_3_CN	51
16	CuCl_2_	CsOH	CH_3_CN	57
17	CuO	CsOH	CH_3_CN	34
18	none	CsOH	CH_3_CN	0
19	CuCl	CsOH	Toluene	31
20	CuCl	CsOH	THF	65
21	CuCl	CsOH	PhCl	36
22	CuCl	CsOH	DMF	22

*^a^* *Reaction conditions*: **1a** (0.2 mmol), **2a** (0.3 mmol), CAN (0.3 mmol), Cu source (10 mol%), 2,2'-bipyridine (10 mol%), TEMPO (10 mol%), base (0.5 mmol), and solvent (2 mL), O_2_, 30 °C, 24 h then 60 °C for 24 h. *^b^* Isolated yield of **3a**.

Screening revealed that the use of CsOH or KOH as a base achieved the best results. Other bases, including KF, Li_2_CO_3_, CsOAc, K_2_CO_3_, K_3_PO_4_, *t*-BuOK, LiOH, and NaOH, were less efficient (Table 1, entries 2–10). Among the copper sources used (e.g., CuOTf, CuBr, CuCl, Cu(OTf)_2_, Cu(OAc)_2_, CuBr_2_, CuCl_2_, and CuO), CuCl exhibited the highest catalytic reactivity (91% yield, Table 1, entries 10–17). No reaction occurred when the procedure was carried out in the absence of copper catalyst (Table 1, entry 18). Finally, we studied the solvent effect and found that CH_3_CN was superior to toluene, THF, PhCl, and DMF (Table 1, entries 12, 19–22). In addition, the reaction failed to give the desired product when the procedure was carried out under a N_2_ atmosphere.

With the optimized reaction conditions in hand, we next explored the substrate scope of (2-aminophenyl)methanols **1** and aldehydes **2** for the one-pot tandem multicomponent cyclization reaction (Table 2). First, the reaction of (2-aminophenyl)methanol (**1a**) with various aldehydes **2a**−**k** in the presence of CAN was investigated under the standard conditions. The results disclosed that a variety of aldehydes are suitable substrates, and the cyclization reactions provide the corresponding products in moderate to good yields (Table 2, entries 1−11).

**Table 2:** Substrate scope of (2-aminophenyl)methanols and aldehydes ^a^. 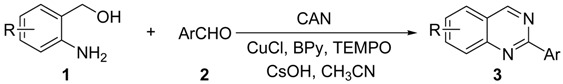

Entry	(2-Aminophenyl)methanols (1)	Aldehydes (2)	Product (3)	Yield (%) ^b^
1	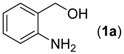	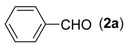	**3a**	91
2	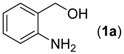	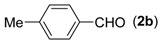	**3b**	84
3	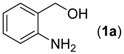	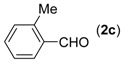	**3c**	66
4	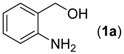	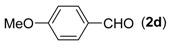	**3d**	82
5	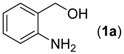	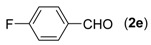	**3e**	93
6	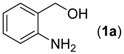	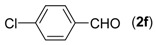	**3f**	86
7	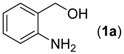	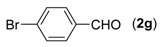	**3g**	84
8	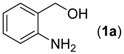	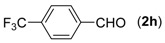	**3h**	71
9	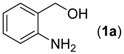	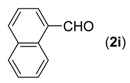	**3i**	78
10	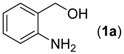	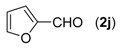	**3j**	87
11	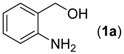	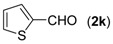	**3k**	89
12	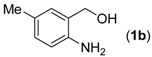	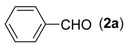	**3l**	87
13	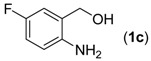	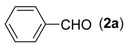	**3m**	81
14	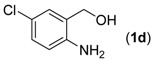	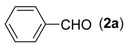	**3n**	80
15	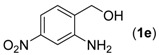	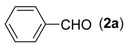	**3o**	67

*^a^* *Reaction conditions*: **1** (0.2 mmol), **2** (0.3 mmol), CAN (0.3 mmol), CuCl (10 mol%), 2,2'-bipyridine (10 mol%), TEMPO (10 mol%), CsOH (0.5 mmol), and CH_3_CN (2 mL), O_2_, 30 °C, 24 h then 60 °C for 24 h. *^b^* Isolated yield.

First, the mono-substituent positions at the aryl moiety of aldehydes were evaluated, and the results demonstrated that steric effects of substituents had some effects on the reaction. For example, when the cyclization reaction of **1a** with *para-*, and *ortho*-methylbenzaldehyde was examined, 84% of **3b** was isolated, while the yield of **3c** decreased to 66% (Table 2, entries 2–3). The electronic properties of the substituents on the phenyl ring of the aldehydes also affected the yields of the reaction to some extent. Generally, the aldehydes bearing an electron-withdrawing substituent (e.g., –F, –Cl and –Br, Table 2, entries 5–7) afforded a slightly higher yield of cyclization products than those analogues bearing an electron-donating substituent (e.g., –Me and –OMe, Table 2, entries 2–4). However, aldehydes bearing a strong electron-withdrawing substituent (e.g.,–CF_3_) at the *para* position afforded a slightly lower yield of product **3h** (Table 2, entry 8). A moderate yield of 2-(naphthalen-2-yl)quinazoline (**3i**) was observed using 1-naphthaldehyde (**2i**) as substrate (Table 2, entry 9). In addition, heterocyclic aldehydes, such as 2-furylaldehyde (**2j**) and 2-thienylaldehyde (**2k**) could also be used as the substrates, leading to the corresponding desired products 2-(furan-2-yl)quinazoline (**3j**) and 2-(thiophen-2-yl)quinazoline (**3k**) in 87% and 89% yields, respectively (Table 2, entries 10–11). 

Subsequently, several (2-aminophenyl)methanols **1b**–**e**, which bear different substituents at the aryl moiety, were evaluated (Table 2, entries 12−15). The results showed that different functional groups, including methyl, fluoro, chloro, and nitro, were well tolerated under the standard conditions. In general, the (2-aminophenyl)methanols bearing an electron-donating substituent produced a slightly higher yield of cyclization products than those analogues bearing an electron-withdrawing substituent. For example, substrates **1b** and **1e**, bearing a methyl or nitro group, reacted with substrate **2a** to give the corresponding products **3l** and **3o** in 87% and 67% yields, respectively (Table 2, entries 12 and 15). It is noteworthy that halogen-containing substrates **1c** and **1d** produced the desired products **3m** and **3n** in 80% and 81% yields, respectively (Table 2, entries 13–14).

**Scheme 2:**
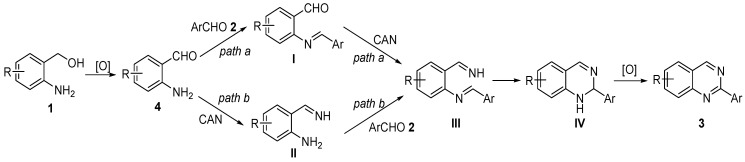
Plausible reaction pathways.

Two plausible reaction pathways for the formation of 2-arylquinazolines have been established and are shown in Scheme 2. First, aerobic alcohol oxidation reaction of (2-aminophenyl)methanols **1** generates 2-aminobenzaldehydes **4**. An imine intermediate III is formed from intermediate I or intermediate II by two plausible reaction pathways, respectively. Then intramolecular cyclization of intermediate III affords dihydroquinazolines IV. Aromatization of intermediate IV gives 2-arylquinazolines 3 as the desired products. However, the mechanism in detail on the formation of the 2-arylquinazolines remains unclear in current stage.

## 3. Experimental

### 3.1. General

Melting points are uncorrected. ^1^H-NMR and ^13^C-NMR spectra were measured on a 500 MHz or 300 MHz spectrometer using DMSO-*d_6_* or CDCl_3_ as the solvent with tetramethylsilane (TMS) as an internal standard at room temperature. Chemical shifts are given n *δ* relative to TMS, and the coupling constants *J* are given in hertz. High-resolution mass spectra were recorded on an ESI-Q-TOF mass spectrometer. Other commercially obtained reagents were used without further purification. All reactions under nitrogen atmosphere were conducted using standard Schlenk techniques. Column chromatography was performed using EM silica gel 60 (300−400 mesh).

### 3.2. General Procedure for the Synthesis of 2-Arylquinazolins

To a Schlenk tube were added (2-aminophenyl)methanols **1** (0.2 mmol), aldehydes **2** (0.3 mmol), CAN (0.3 mmol), CuCl (0.02 mmol), 2,2'-bipyridine (0.02 mmol), TEMPO (0.02 mmol), CsOH (0.5 mmol), and CH_3_CN (2 mL). Next the tube was charged with O_2_ (1 atm), and was stirred constantly at 30 °C for 24 h, then at 60 °C for 24 h. After the completion of the reaction, the reaction mixture was cooled to room temperature, diluted with ethyl acetate, and washed with brine. After the aqueous layer was extracted with ethyl acetate, the combined organic layers were dried over anhydrous MgSO_4_ and evaporated under reduced pressure. The residue was purified by flash column chromatography (hexane/ethyl acetate) to afford the desired products **3**.

*2-Phenylquinazoline* (**3a**) [24]. Pale yellow solid (91% yield), mp 98–99 °C (Lit. 97–98 °C); ^1^H-NMR (DMSO-*d_6_*, 300 MHz): δ 9.70 (s, 1H), 8.55–8.59 (m, 2H), 8.17 (d, *J* = 8.1 Hz, 1H), 8.00–8.08 (m, 2H), 7.71–7.76 (m, 1H), 7.55–7.59 (m, 3H); ^13^C-NMR (DMSO-*d_6_*, 75 MHz): δ 161.3, 159.8, 149.8, 137.4, 134.8, 130.8, 128.7, 128.1, 127.9, 127.8, 123.3.

*2-p-Tolylquinazoline* (**3b**) [24]*.* Yellow solid (84% yield), mp 109–111 °C (Lit. 107–109 °C); ^1^H-NMR (CDCl_3_, 500 MHz): δ 9.44 (s, 1H), 8.52 (d, *J* = 8.0 Hz, 2H), 8.07 (d, *J* = 8.0 Hz, 1H), 7.87–7.90 (m, 2H), 7.56–7.60 (m, 1H), 7.35 (d, *J* = 8.0 Hz, 2H), 2.45 (s, 3H); ^13^C-NMR (CDCl_3_, 125 MHz): δ 161.1, 160.4, 150.8, 140.8, 135.3, 134.0, 129.4, 128.52, 128.50, 127.1, 127.0, 123.5, 21.5.

*2-o-Tolylquinazoline* (**3c**) [25]*.* Colorless oil (66% yield); ^1^H-NMR (CDCl_3_, 500 MHz): δ 9.49 (s, 1H), 8.11 (d, *J* = 8.5 Hz, 1H), 7.89–7.95 (m, 2H), 7.77 (d, *J* = 7.5 Hz, 1H), 7.63 (t, *J* = 7.5 Hz, 1H), 7.44 (t, *J* = 7.5 Hz, 1H), 7.05–7.11 (m, 2H), 3.87 (s, 3 H); ^13^C-NMR (CDCl_3_, 125 MHz): δ 162.4, 160.0, 157.7, 150.6, 134.0, 131.7, 130.8, 129.0, 128.5, 127.5, 127.0, 123.1, 120.7, 111.9, 56.0.

*2-(4-Methoxyphenyl)quinazoline* (**3d**) [24]. White solid (82% yield), mp 93–94 °C (Lit. 91–93 °C); ^1^H-NMR (CDCl_3_, 500 MHz): δ 9.40 (s, 1H), 8.57–8.59 (m, 2H), 8.03 (d, *J* = 8.9 Hz, 1H), 7.86 (t, *J* = 9.7 Hz, 2H), 7.55 (t, *J* = 8.0 Hz, 1H), 7.04 (d, *J* = 9.0 Hz, 2H), 3.89 (s, 3H); ^13^C-NMR (CDCl_3_, 125 MHz): δ 161.9, 160.9, 160.4, 150.9, 134.0, 130.9 130.2, 128.4, 127.1, 126.8, 123.3, 114.0, 55.4.

*2-(4-Fluorophenyl)quinazoline* (**3e**) [24]. White solid (93% yield), mp 138–139 °C (Lit. 135–137 °C); ^1^H-NMR (CDCl_3_, 500 MHz): δ 9.42 (s, 1H), 8.61–8.64 (m, 2H), 8.06 (d, *J* = 8.5 Hz, 1H), 7.89 (t, *J* = 8.0 Hz, 2H), 7.60 (t, *J* = 7.0 Hz, 1H), 7.20 (t, *J* = 8.5 Hz, 2H); ^13^C-NMR (CDCl_3_, 125 MHz): δ 165.6, 163.7, 160.5, 160.1, 150.7, 134.1, 130.7, 130.6, 128.5, 127.2, 127.1, 123.5, 115.6, 115.4.

*2-(4-Chlorophenyl)-quinazoline* (**3f**) [24]. White solid (86% yield), mp 135–137 °C (Lit. 133–135 °C); ^1^H-NMR (DMSO-d_6_, 500 MHz): δ 9.72 (s, 1H), 8.57 (d, *J* = 8.5 Hz, 2H), 8.19 (d, *J* = 8.0 Hz, 1H), 8.04–8.08 (m, 2H), 7.75–7.78 (m, 1H ), 7.64 (d, *J* = 8.5 Hz, 2H); ^13^C-NMR (DMSO-d_6_, 125 MHz): δ 161.5, 158.8, 149.8, 136.3, 135.8, 135.1, 129.9, 128.9, 127.93, 127.90, 123.4.

*2-(4-Bromophenyl)quinazoline* (**3g**) [23]. White solid (84% yield), mp 121–123 °C (Lit. 120–121 °C); ^1^H-NMR (CDCl_3_, 500 MHz): δ 9.43 (s, 1H), 8.50 (d, *J* = 8.5 Hz, 2H), 8.06 (d, *J* = 9.0 Hz, 1H), 7.89–7.92 (m, 2H), 7.60–7.66 (m, 3H); ^13^C-NMR (CDCl_3_, 125 MHz): δ 160.5, 160.1, 150.7, 137.0, 134.2, 131.8, 130.2, 128.6, 127.4, 127.1, 125.4, 123.6.

*2-(4-(Trifluoromethyl)phenyl)quinazoline* (**3h**) [23]. White solid (71% yield), mp 144–146 °C (Lit. not reported); ^1^H-NMR (DMSO-*d_6_*, 500 MHz): δ 9.76 (s, 1H), 8.75 (d, *J* = 8.5 Hz, 2H), 8.21 (d, *J* = 8.0 Hz, 1H), 8.06–8.12 (m, 2H), 7.93 (d, *J* = 8.5 Hz, 2H), 7.78–7.81 (m, 1H); ^13^C-NMR (DMSO-*d_6_*, 125 MHz): δ 161.6, 158.4, 149.7, 141.2, 135.1, 130.7, 150.5, 128.7, 128.4, 128.0, 127.9, 125.8, 125.7, 125.3, 123.6, 123.1.

*2-(Naphthalene-1-yl)quinazoline* (**3i**) [25]. white solid (78% yield), mp 121–123 °C (Lit. 120–121 °C); ^1^H-NMR (CDCl_3_, 500 MHz): δ 9.60 (s, 1H), 8.70 (d, *J* = 8.0 Hz, 1H), 8.17–8.19 (m, 2H), 7.93–8.03 (m, 4H), 7.70 (t, *J* = 7.5 Hz, 1H), 7.64 (t, *J* = 7.5 Hz, 1H), 7.52-7.58 (m, 2H); ^13^C-NMR (CDCl_3_, 125 MHz): δ 163.5, 160.4, 150.6, 136.3, 134.3, 134.2, 131.2, 130.4, 129.6, 128.7, 128.5, 127.7, 127.1, 126.8, 125.89, 125.87, 125.3, 123.1.

*2-(Furan-2-yl)quinazoline* (**3j**) [26]. Brown solid (87% yield), mp 132–134 °C (Lit. 131–132 °C); ^1^H-NMR (CDCl_3_, 500 MHz): δ 9.36 (s, 1H), 8.08 (d, *J* = 9.0 Hz, 1H), 7.87–7.90 (m, 2H), 7.68 (s, 1H), 7.60 (t, *J* = 6.0 Hz, 1H), 7.44–7.45 (m, 1H), 6.60–6.61 (m, 1H); ^13^C-NMR (CDCl_3_, 125 MHz): δ 160.7, 154.1, 152.5, 150.4, 145.3, 134.5, 128.4, 127.2, 123.4, 114.1, 112.3.

*2-(Thiophene-2-yl)quinazoline* (**3k**) [13]. White solid (89% yield), mp 132–133 °C (Lit. 132–134 °C); ^1^H-NMR (CDCl_3_, 500 MHz): δ 9.34 (s, 1H), 8.14–8.15 (m, 1H), 8.00 (d, *J* = 9.0 Hz, 1H), 7.85–7.88 (m, 2H), 7.51–7.57 (m, 2H), 7.18–7.20 (m, 1H); ^13^C-NMR (CDCl_3_, 125 MHz): δ 160.5, 157.9, 150.6, 143.8, 134.3, 129.9, 129.2, 128.3, 128.2, 127.2, 127.0, 123.4.

*6-Methyl-2-phenylquinazoline* (**3l**) [27]. Pale yellow solid (87% yield), mp 131–132 °C (Lit. 130–132 °C); ^1^H-NMR (CDCl_3_, 500 MHz): δ 9.38 (s, 1H), 8.60 (d, *J* = 8.5 Hz, 2H), 7.98 (d, *J* = 8.5 Hz, 1H), 7.72–7.74 (m, 1H), 7.67 (s, 1H), 7.50–7.55 (m, 3H), 2.58 (s, 3H); ^13^C-NMR (CDCl_3_, 125 MHz): δ 160.4, 159.7, 149.4, 138.2, 137.4, 136.4, 130.4, 128.6, 128.4, 128.3, 125.8, 123.6, 21.6.

*6-Fluoro-2-phenylquinazoline* (**3m**) [14]. White solid (81% yield), mp 121–122 °C (Lit. 120–121 °C); ^1^H-NMR (CDCl_3_, 500 MHz): δ 9.43 (s, 1H), 8.59–8.61 (m, 2H), 8.09–8.11 (m, 1H), 7.65–7.70 (m, 1H), 7.51–7.55 (m, 4H); ^13^C-NMR (CDCl_3_, 125 MHz): δ 161.4, 160.7, 159.8, 159.7, 159.4, 148.0, 128.2, 137.7, 131.4, 131.3, 130.7, 128.7, 128.5, 124.6, 124.4, 124.0, 123.9, 110.2, 110.0.

*6-Chloro-2-phenylquinazoline* (**3n**) [17]. Pale yellow solid (80% yield), mp 158–159 °C (Lit. 157–159 °C); ^1^H-NMR (CDCl_3_, 500 MHz): δ 9.38 (s, 1H), 8.59–8.61 (m, 2H), 8.02 (d, *J* = 9.0 Hz, 1H), 7.89 (d, *J* = 2.5 Hz, 1H), 7.81–7.83 (m, 1H), 7.51–7.55 (m, 3H); ^13^C-NMR (CDCl_3_, 125 MHz): δ 161.3, 159.5, 149.2, 137.6, 135.0, 132.8, 130.9, 130.4, 128.7, 128.6, 125.8, 124.0.

*7-Nitro-2-phenylquinazoline* (**3o**)*.* White solid (67% yield), mp 142–144 °C; IR (KBr) 2925, 1687, 1567, 1526, 1421, 1326, 1291, 927, 823, 740, 706, 686, 668 cm^−1^; ^1^H-NMR (CDCl_3_, 500 MHz): δ 9.62 (s, 1H), 8.97 (d, *J* = 2 Hz, 1H), 8.64–8.66 (m, 2H), 8.357–8.361 (m, 1H), 8.11 (d, *J* = 8.5 Hz, 1H), 7.56–7.58 (m, 3 H); ^13^C-NMR (CDCl_3_, 125 MHz): δ 162.9, 160.8, 151.2, 150.5, 136.9, 131.6, 129.0, 128.9, 128.8, 125.7, 124.9, 120.7. HRMS (ESI) *m/z*: [M+H]^+^ Calcd for C_14_H_10_N_3_O_2_ 251.0695; Found: 251.0681.

## 4. Conclusions

In summary, we have successfully developed a new protocol for the copper-catalyzed cascade reaction of (2-aminophenyl)methanols, aldehydes, and ceric ammonium nitrate to afford a series of 2-arylquinazolines in moderate to excellent yields. Further efforts to extend this catalytic system to the preparation of other useful heterocyclic compounds are currently underway in our laboratories.
